# Limb Salvage Strategy Amendment for a Better Future in the Era of Bone Cancer Therapy: A Cross-Sectional Study in North India

**DOI:** 10.7759/cureus.41768

**Published:** 2023-07-12

**Authors:** Bhavesh Kumar, Priyanka Sharma, Kumar Shantanu, Sanjiv Kumar, Rishabh Agarwal, Ashish Kumar, Deepak Kumar

**Affiliations:** 1 Orthopaedic Surgery, King George's Medical University, Lucknow, IND; 2 Pathology, King George's Medical University, Lucknow, IND

**Keywords:** ewing sarcoma, amputation, north indian, megaprosthesis, giant cell tumour (gct), osteosarcoma, hui -3 index, sf-36 score, quality of life

## Abstract

Background: Bone tumors remain a formidable challenge for orthopedic surgeons. In developing countries, the challenge is exacerbated by limited diagnostic, therapeutic, and management facilities and ignorance. Patients with upper and lower-extremity muscle and skeletal tumors are candidates for amputation or surgical rescue of the limbs. Traditionally, limb rescue surgery by neo-adjuvant chemotherapy is the preferred surgery method for localized carcinoma. Amputations are usually reserved for patients with increased tumor size. The purpose of this study is to investigate health-related quality of life (HRQOL) and physical disability, focusing on surgical care, gender, and age, in adolescent and young adult survivors of malignant bone tumors treated surgically.

Methods: This cross-sectional study consists of 38 long-term survivors who underwent amputation or limb-salvage surgery at King George's Medical University, Lucknow, from 2019 to 2022. After obtaining ethical clearance and informed consent, 38 patients which included 26 patients treated with limb salvage in Group A and 12 patients treated with amputation in Group B were included in the study. The SF-36 and HUI3 scores were used to assess the functional outcome and health-related QoL of these patients.

Results: After minimal six months of interventions, we have found a significant improvement in all the following factors: physical functioning (P=0.000), role limitations due to physical health (P=0.000) and emotional problems (P=0.001), energy/fatigue (P=0.000), emotional well-being (P=0.000), social functioning (P=0.000), pain (P=0.000), and general health (P=0.000). Group A showed a higher degree of significance than Group B through SF-36 (Short Form-36, patient-reported outcome), whereas HUI-3 did not show any significant outcomes (P=0.347).

Conclusion: The overall quality of life of patients with salvaged limbs appears to be higher than that of the quality of life of amputee patients in tumor survivor patients. Further analyses must be carried out to verify the results and focus on areas that have a major impact on the overall quality of life using other assessment tools. The impact of therapy on the quality of life depends on maintaining the necessary structures for functional functions, adjusting patient expectations to cancer treatments, and designing long-term rehabilitation programs to support functional functions.

## Introduction

Osteosarcoma, Ewing sarcoma, and rhabdomyosarcoma are the most common malignant musculoskeletal tumors in children and adolescents [1]. Bone tumors account for only a small proportion of all malignancies diagnosed globally. Osteosarcoma and Ewing sarcoma are two major tumors that occur during adolescent growth spurts [1]. Elderly people also experienced small incidence peaks. Over 40% of osteosarcoma and Ewing sarcoma cases occur during adolescence, especially in the second decade of life [1]. Malignant bone tumors diagnosed at 20 years account for 6% of all malignancies [2-4]. Both malignant bone tumors prefer long bones, such as the femur, pelvis, and humerus [5,6]. These malignant bone tumors in the girdle and extremities pose a very challenging treatment issue [6-9]. Globally, limb salvage and multi-agent chemotherapy are the standard treatments for osteosarcoma. Plenty of research reports a 50%-60% five-year survival rate for osteosarcoma [6-9]. Limb salvage therapy can increase survival rates by up to 90%, according to numerous studies at specialized centers [7]. Despite the presence of these surgical approaches that enhance life expectancy, there is still a need for further research to assess the quality of life after surgical treatment [10]. Previous studies employed the SF-36 questionnaire and HRQOL to assess clinical measures; however, it is unknown whether health-related quality of life (HRQOL) has improved significantly [11]. In addition, it is necessary to know which domains of HRQOL are low among patients after surgery compared to the general population. Until now, no study has compared HRQOL after bone tumor surgery with the general population. The Indian population has still not found evidence. The present study was carried out to quantify and compare the quality of life in patients with malignant bone tumors treated surgically through different surgical interventions including limb salvage therapy. Management of malignant bone tumors in the girdle and extremities poses a very challenging treatment issue. Since the advent of diagnostic and chemotherapeutic approaches over the last few decades, limb salvage has replaced amputation. Throughout the developed world, there has been an increase in the evaluation of functional and oncological outcomes associated with the surgical treatment of osteosarcoma. However, developed countries have lagged behind in this regard. However, evaluation of functional outcomes after surgical treatment is a crucial consideration in the management of malignant tumors of the extremities and girdle. Currently, there are very few studies on the contemporary treatment of osteosarcoma conducted within a single institution in Indian patients. Hence, the purpose of this study was to compare the quality of life of survivors who underwent amputation or limb-preservation procedures for bone sarcoma [12].

## Materials and methods

The current cross-sectional study was carried out from July 2021 to 2023 in OPD, and we have recruited patients managed from January 2017 to December 2021 in the Department of Orthopaedics at KGMU (King George Medical University), Lucknow after obtaining ethical approval from the Institutional Ethics Committee (859/Ethics/2022 and Ref. code XI-PGTSC-IIA/P19). Thirty-eight patients (26 patients treated with limb salvage in Group A and 12 patients treated with amputation in Group B) who had been diagnosed with malignant bone tumors were included as per the inclusion criteria with written informed consent. Treatment decision was purely based on the post-chemo status of a tumor, if non-responsive to chemotherapy and extracompartmental in nature would be considered for amputation. Any tumor that is chemoresponsive and not encasing the neurovascular structures would be considered for limb salvage. In the outpatient clinics, after precise history taking, physical examination, and rechecking the previous demographic and medical data, the 36-Item Short Form Survey (SF-36) and Health Utility Index Mark 3 (HUI-3) were administered to the patients. All surgical procedures were performed in the Department of Orthopaedics, KGMU, Lucknow. Inclusion criteria were patients with malignant bone tumors managed surgically between January 2017 and December 2021, cases of either gender of age 9-59 years, patients diagnosed with any type of malignant bone tumor who had completed chemotherapy and been treated surgically including GCT campanacci grade III with malignant changes with a post-operative period of more than six months. Exclusion criteria were patients with incomplete HRQOL questionnaire data and incomplete diagnosis/treatment details. Quality-of-life outcomes were recorded post-operatively at least after six months. There were 38 patients evaluated pre- and post-surgically from SF-36 and HUI-3 index scores.

Statistical analysis 

The data were entered into the MS Excel Spreadsheet, and the analysis was done using IBM SPSS Statistics for Windows, Version 26 (Released 2019; IBM Corp., Armonk, New York, United States). The mean, standard deviation, and median were calculated to represent continuous variables. Quantitative variables were compared using an unpaired t-test between two groups and a paired t-test between two groups, Group A and Group B. We have found a more significant p value for intra-group analysis between pre-operative surgical analysis and post-operative surgical analysis using SF-36. Physical functioning (PF), role limitations due to physical health (RLPH) and emotional problems (RLEP), energy/fatigue (E/F), emotional well-being (EWB), social functioning, pain (P), and general health (GH) showed p=0.19, p= not showed any result, p=not showed any result, p= 0.793, p= 0.419, p= 0.002, p=0.002, and p=0.519 in Group 1 (pre-op) and p=0.000, p=0.028, p=0.227, p=0.002, p= 0.003, p=0.150, p= 0.070, and p=0.000 in Group 2, respectively. HUI-3 index quality-of-life assessment tool did not show any significant differences during intra-analysis, p=0.162 and p=0.248 respectively. In inter-group analysis, more highly significant quality of life was also found between post-operative and pre-operative analyses using SF-36 scores except physical functioning, emotional health, and pain. PF, RLPH and RLEP, E/F, EWB, social functioning, P, and GH showed p=0.0001, p=0.000, p=0.001, p= 0.000, p= 0.000, p= 0.000, p=0.000, and p=0.000 in Group 1 (pre-op) and p=0.0008, p=0.000, p=0.002, p=0.000, p= 0.002, p=0.000, p= 0.002, and p=0.000 in Group 2, respectively, while quality of life using the HUI-3 index showed no significant differences between pre-operative and post-operative analyses in inter-group analysis p=0.521 and p=0.347 in Group 1 and Group 2, respectively.

**Figure 1:**
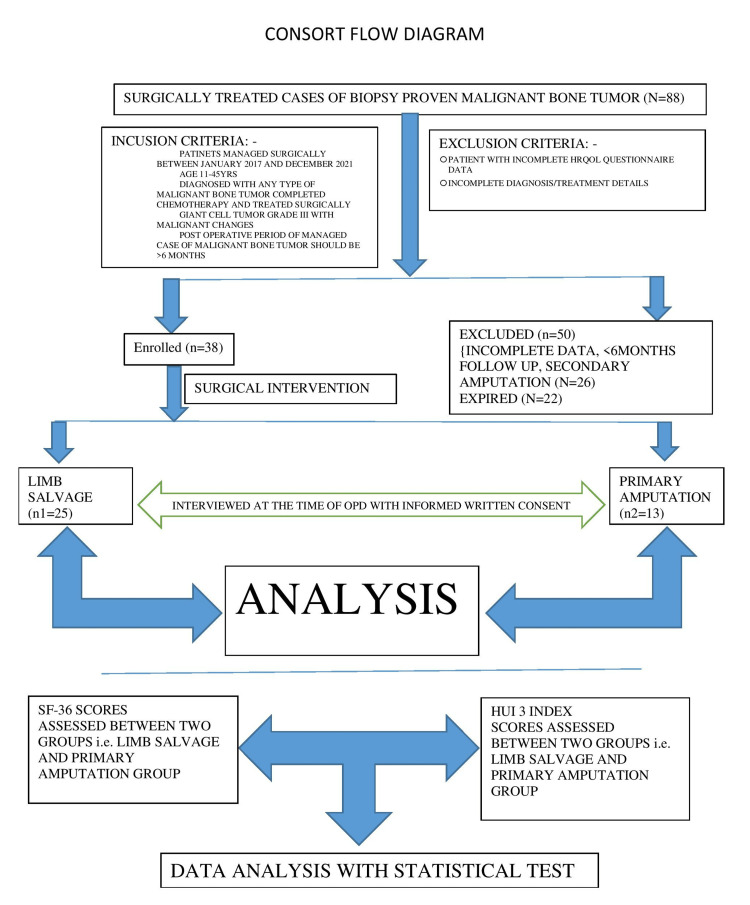
Consort flow diagram

Figure 1 depicts the consort summary of the work.

## Results

The study population consisted of 38 study subjects aged between 9 and 59 years, including three groups: 9-15 years, 16-30 years, and 31-59 years. The majority of patients (81.58%, n=31) were between 11 and 30 years old at the time of diagnosis (Figure 1). A significantly higher percentage of males (60.52%, n=23) were affected by malignant bone tumors than females (39.48%, n=15) (Table 1). A significant percentage of patients were diagnosed with osteosarcoma (65.79%, n=25) followed by giant cell tumors (grade III) (23.68%, n=9) and Ewing sarcoma (10.53%, n=4) (Table 2). Malignant tumors affect both the left (50%, n=19) and right (50%, n=19) sides in equal proportion (Table 3). A significant proportion of proximal and distal femurs (44.74%, n=17) was observed among patients, followed by proximal and distal tibia (36.84%, n=14) and proximal and distal humerus (18.42%, n=7) (Table 4). The majority of patients were managed through limb salvage therapy (67.59%, n=25) while 13 patients (34.21%) were managed through amputation (Table 5). In the case of limb salvage therapy, megaprosthesis was performed in 60.52% (n=23) of patients, whereas both autograft and rotationplasty were performed in 2.63% (n=1) and 2.63% (n=1) patients, respectively (Table 5). The majority of amputations were performed above the knee (21.06%, n=8) followed by below the knee (10.53, n=4) and through shoulder disarticulation (2.63%, n=1) (Table 5). Males with osteosarcoma made up most cases (60.53%, n=23) than females (5.23, n=2) (Table 5). Cases of giant cell tumors (grade III) (23.68%, n=9) and Ewing sarcoma (10.53%, n=4) were only observed in females (Table 2). The mean subscale items i.e. PH, RLEP, E/F, EWB, SF, P, and GH of the participants in the limb salvage group within pre-operatively vs post-operatively p-values were higher than those of the amputee patients, which was statistically significant with a p-value of <0.05 (Tables 6, 7) using SF-36 scores. Non-significant comparative analysis was observed for both inter-group (p=0.347) and intra-group (p=0.248) patients with limb salvage therapy and amputation (Tables 8, 9) using the HUI-3 index.

**Table 1:** Gender distribution in the study population

Gender	Frequency	Percentage
Male	23	60.52
Female	15	39.48
Total	38	100.0

**Table 2:** Diagnosis of the study population at admission

Diagnosis	Frequency	Percentage
Giant cell tumor (Grade III)	9	23.68
Osteosarcoma	25	65.79
Ewing sarcoma	4	10.53
Total	38	100

**Table 3:** Side distribution in the study population

Side	Frequency	Percentage
Left	19	50
Right	19	50
Total	38	100

**Table 4:** Site distribution in the study population

Site distribution	Frequency	Percentage
Proximal and distal femur	17	44.74
Proximal and distal humerus	7	18.42
Proximal and distal tibia	14	36.84
Total	38	100

**Table 5:** Management distribution in the study population

Management	The type of management	Frequency	Percentage
	Megaprosthesis	23	60.52
Limb salvage	Autograft	1	2.63
	Rotationplasty	1	2.63
	The above knee	8	21.06
Amputation	Below knee	4	10.53
	Through shoulder disarticulation	1	2.63
	Total	38	100

**Table 6:** Intra-group comparative analysis of pre-surgical (Group 1) and post-surgical (limb salvage and amputation) (Group 2) periods of patients using SF-36 scores. Statistical significance shows a p-value less than 0.05. * noted as less significant; ** noted as moderately significant, and *** noted as highly significant.

S. No.	Scale Items	Group 1	Group 1	p-value	Group 2	Group 2	p-value
1.	Physical Function	Pre-op	Pre-op	0.19	Post-op	Post-op	0.000***
		Mean±SD	Mean±SD		Mean±SD	Mean±SD	
		17.50±18.67	10.00±8.79		74.04±17.38	39.58±29.42	
		95% CI			95% CI		
		-4.04 to 19.04			19.04 to 49.8		
2.	Role limitation due to physical health	Pre-op	Pre-op	--	Post-op	Post-op	0.028*
		Mean±SD	Mean±SD		Mean±SD	Mean±SD	
		.00±00	.00±.00		58.65±17.23	43.75±21.65	
		95% CI			95% CI		
		--			1.67 to 28.14		
3.	Role limitation due to emotional problems	Pre-op	Pre-op	--	Post-op	Post-op	0.227
		Mean±SD	Mean±SD		Mean±SD	Mean±SD	
		.00±00	.00±.00		37.18±48.37	58.33±51.49	
		95% CI			95% CI		
		--			-56.08 to 13.77		
4.	Energy/ fatigue	Pre-op	Pre-op	0.793	Post-op	Post-op	0.002**
		Mean±SD	Mean±SD		Mean±SD	Mean±SD	
		16.53±3.68	17.08±9.16		83.65±11.96	64.58±24.26	
		95% CI			95% CI		
		-4.73 to 3.64			7.24 to 30.89		
5.	Emotional well-being	Pre-op	Pre-op	0.419	Post-op	Post-op	0.003**
		Mean±SD	Mean±SD		Mean±SD	Mean±SD	
		36.0±7.07	33.33± 13.14		87.69±10.85	69.33±24.44	
		95% CI			95% CI		
		-3.95 to 9.28			6.85 to 29.86		
6.	Social functioning	Pre-op	Pre-op	0.002**	Post-op	Post-op	0.150
		Mean±SD	Mean±SD		Mean±SD	Mean±SD	
		37.50±3.53	30.20±9.91		86.06±13.38	78.13±19.31	
		95% CI			95% CI		
		2.88 to 11.69			-2.99 to 18.85		
7.	Pain	Pre-op	Pre-op	0.002**	Post-op	Post-op	0.070
		Mean±SD	Mean±SD		Mean±SD	Mean±SD	
		52.98±13.67	37.08		78.46±9.33	69.38±20.95	
		95% CI			95% CI		
		6.03 to 25.76			-.78 to 18.95		
8.	General Health	Pre-op	Pre-op	0.519	Post-op	Post-op	0.000***
		Mean±SD	Mean±SD		Mean±SD	Mean±SD	
		4.81±4.12	3.75±5.69		86.73±12.56	59.17±28.98	
		95% CI			95% CI		
		-2.24 to 4.35			14.02 to 41.11		

**Table 7:** Inter-group comparative analysis between limb salvage (Group 1) and amputation (Group 2) patients in pre-operative and post-operative periods using SF-36 scores. Statistical significance shows a p-value less than 0.05. * Noted as less significant; ** noted as moderately significant, and *** noted as highly significant.

S. No.	Scale Items	Group 1		p-value	Group 2			p-value
1.	Physical Function	Pre-op	Post-op	0.000**	Pre-op		Post-op	0.008
		Mean±SD	Mean±SD		Mean±SD		Mean±SD	
		17.50±18.67	74.04±17.38		10±8.79		39.58±29.42	
		95% CI			95% CI			
		-67.92 to -45.16			-49.51 to -9.65			
2.	Role limitation due to physical health	Pre-op	Post-op	0.000**	Pre-op		Post-op	0.000**
		Mean±SD	Mean±SD		Mean±SD		Mean±SD	
		.00±.00	58.65±17.24		.00±.00		43.75±21.65	
		95% CI			95% CI			
		-65.61 to -51.69			-57.51 to -29.99			
3.	Role limitation due to emotional problems	Pre-op	Post-op	0.001**	Pre-op	Post-op		0.002**
		Mean±SD	Mean±SD		Mean±SD	Mean±SD		
		.00±.00	37.18±48.37		.00±.00	58.33±51.49		
		95% CI			95% CI			
		-56.72 to -17.64			-91.05 to -25.62			
4.	Energy/fatigue	Pre-op	Post-op	0.000***	Pre-op		Post-op	0.000***
		Mean±SD	Mean±SD		Mean±SD		Mean±SD	
		16.54±3.67	83.65±11.96		17.08±9.16		64.58±24.26	
		95% CI			95% CI			
		-72.42 to -61.81			-63.03 to -31.96			
5.	Emotional well-being	Pre-op	Post-op	0.000***	Pre-op		Post-op	0.002**
		Mean±SD	Mean±SD		Mean±SD		Mean±SD	
		36.00±7.06	87.69±10.85		33.33±13.14		69.33±24.44	
		95% CI			95% CI			
		-57.32 to 46.06			-55.42 to -16.58			
6.	Social functioning	Pre-op	Post-op	0.000***	Pre-op		Post-op	0.000**
		Mean±SD	Mean±SD		Mean±SD		Mean±SD	
		37.50±3.53	86.06±13.38		30.21±9.91		78.12±19.31	
		95% CI			95% CI			
		-54.15 to -42.97			-63.74 to 32.09			
7.	Pain	Pre-op	Post-op	0.000***	Pre-op		Post-op	0.002**
		Mean±SD	Mean±SD		Mean±SD		Mean±SD	
		52.98±13.67	78.46±9.33		37.08±14.53		69.37±20.95	
		95% CI			95% CI			
		-32.62 to -18.34			-49.83 to -14.75			
8.	General Health	Pre-op	Post-op	.000***	Pre-op		Post-op	.000***
		Mean±SD	Mean±SD		Mean±SD		Mean±SD	
		4.80±4.12	86.73±12.56		3.75±5.69		59.16±28.98	
		95% CI			95% CI			
		-87.66 to -76.18			-74.26 to -36.57			

**Table 8:** Intra-group comparative analysis of pre-surgical (Group 1) and post-surgical (limb salvage and amputation) (Group 2) periods of managed patients using HUI-3 index scores. Statistical significance shows a p-value less than 0.05. * Noted as less significant; ** noted as moderately significant; and *** noted as highly significant.

S. No.	Scale Items	Group 1	Group 2	p- value	Group 1	Group 2	p- value
1.	Overall Health Outcome	Pre-op	Pre-op	0.162	Post-op	Post-op	0.248
		Mean±SD	Mean±SD		Mean±SD	Mean±SD	
		2.07±8.45	41.39±142.27		.99±.01	1.04±.20	
		95% CI			95% CI		
		-95.21 to 16.57			-.12 to .033		

**Table 9:** Inter-group comparative analysis of limb salvage (Group 1) and amputation (Group 2) patients in pre-operative and post-operative periods using the HUI-3 index score. Statistical significance shows a p-value less than 0.05. * noted as less significant; ** noted as moderately significant, and *** noted as highly significant.

S. No.	HUI 3 scale	Group 1		p-value	Group 2		p-value
1.	Overall health outcome	Pre-op	Post-op	0.521	Pre-op	Post-op	0.347
		Mean±SD	Mean±SD		Mean±SD	Mean±SD	
		2.07±8.45	.99±.01		41.39±142.27	1.04±.20	
		95% CI			95% CI		
		-2.33 to 4.49			-50.06 to 130.76		

## Discussion

As a result of recent advances and an increase in the survival rate of bone tumor patients, the use of subjective measurements such as functional score scales and quality-of-life questionnaires has received more attention in clinical studies. This study aims to elucidate the better surgical intervention for patients with malignant bone tumors in adolescents and young adults using SF36 scores and HUI-3 index in terms of PROM and HRQOL. The results of HRQOL measures have shown that SF-36 scores are a reliable and effective measure for Indian osteosarcoma patients. Our data suggest that using the SF-36 scores the mean subscale items, i.e. PH, RLEP, RLPH, E/F, EWB, SF, P, and GH of intra-group analysis within the pre-operative p value versus the post-operative p value were statistically significant with the exception of social function, role of emotional function, and pain, with p values of 0.150, 0.227, and 0.070, respectively [5]. HUI-3 results showed no statistically significant difference in intra-group analysis (Table 8). However, in the inter-group analysis, Limb Recovery Group's responses to subscales of the SF-36 scoring, such as PF, RLPH, RLEP, E/F, EWB, SF, P, and GH, showed a significant improvement after six months and showed a higher degree of significance with a value of less than 0.05 (Table 7). There were no significant differences between Groups 1 and 2 in the HUI-3 index results for overall health results (Table 9). In 2000, Renard et al. evaluated 40 patients with osteosarcoma who underwent either limb rescue surgery or ablative therapy using the American Musculoskeletal Tumor Society Rating Scale (MSTS). The functional results were significantly better after the removal of the limbs compared to ablative therapy, but complications were more common after the limb removal surgery [12]. In 2012, Mavrogenis et al. compared the survival, local recurrence, function, and complications of patients with osteosarcoma of the distal tibia treated with limb recovery or amputation. Survival, local recurrence, and complications were similar, but the function was better in patients who had recovered limbs [13]. In 2007, Yonemoto and colleagues studied the educational and employment status of survivors of long-term high-level osteosarcoma. The proportion of people attending university was higher in the limb-salvage group; however, the functional status and the employment status between the amputation and limb-sparing groups were not significantly different [14]. Robert and his colleagues have studied survivors of osteosarcoma after amputation and rescue of the limbs. Both groups compared the quality of life, body image, self-esteem, and social support. Although there are differences in some areas, none of these differences are statistically significant [15]. Recent systematic reviews and meta-analysis have shown that the outcomes of functional and quality of life in patients with malignant bone tumors in the lower extremities who have undergone limb-saving procedures and amputations are similar [16]. There seems to be more studies, but since these two methods do not have similar results in function score and quality of life, the differences in the basic characteristics of the population analyzed must be carefully expressed. On the other hand, we have used the HUI-3 index (Health Utility Index) along with the SF36 scale to provide a compact but comprehensive framework for describing health conditions. The multi-attribute utility function provides all the information needed to calculate a single summary of health-related quality of life scores (HRQL) for each health state defined by the classification system. The use of HUI is illustrated in clinical trials for many diseases in many countries. HUI provides a comprehensive, reliable, reactive, and effective HRQL measurement of the health status of clinical patients. The overall HRQL utility score for patients is also used for cost-benefit analysis and cost-efficiency analysis. Population norm data are obtained from numerous large-scale general population surveys. The widespread use of HUI facilitates the interpretation of results, allowing comparisons of disease and treatment results and long-term sequels at local, national, and international levels [17]. We also analyzed for the first time two HRQOLs, SF-36 and HUI-3, comparing bone tumor patients in northern India, with significant differences in the use of SF-36 after treatment, but did not show significant changes in HUI-3. This study improved the effectiveness of limb rescue therapy, using the scores of the SF-36 and HUI-3 indexes as widely used questionnaires, and opened up new opportunities for further verification and improvement in quality of life.

Limitations 

The limitations of our study include the following: small sample size, difference in disease status, and lack of a reference group. First, different disease statuses, such as remission, relapse, and complications, can have a major impact on HRQoL. However, the influence of these factors could not be analyzed in this study because of the small sample size. Second, we did not include a reference group to compare the effects of surgery on bone tumors. We used a specific measure with acceptable reliability and validity for the Indian population. For comparison, we used a questionnaire in the general population; however, a more comparable group is needed for further investigation. In our study, we did not follow this management approach because of surgical limitations.

## Conclusions

 Analysis of this study showed that the limb salvage surgery has a higher impact on patient performance and quality of life (QOL) than amputation. In intra group analysis, we have found no significant differences for Role limitations due to emotional problems, social functioning and pain in subscales items of SF-36. While, In Intergroup analysis, we have found higher significant differences in all subscales items in limb salvage group using SF-36 score and showing improved quality of life. however, HUI did not show any significant differences. These results also show that several different aspects of the Heath Related Quality of life (HRQOL) function have been affected, and it might be advisable to include objective measures in postoperative assessments using multiple rating systems tools and a longer follow-up study. The results measures used in this study have been easily administered, inexpensive and can be implemented in clinical practice. Studies are needed to measure subjective and objective physical and mental function before and after surgery in large population samples. Other reconstruction techniques such as extracorporeal radiotherapy and allografts are not considered; therefore, more studies are needed with more patients treated with other modalities. Repetitive measures over about five years could inform us about the course of physical and mental function, enable comparisons with population standards and help to better design targeted and timely postoperative interventions.
